# *Alpinia oxyphylla* Fruit Extract Ameliorates Experimental Autoimmune Encephalomyelitis through the Regulation of Th1/Th17 Cells

**DOI:** 10.1155/2019/6797030

**Published:** 2019-03-14

**Authors:** Kuo-Kuei Huang, Meng-Nan Lin, Yi-Ling Hsu, I-Huang Lu, I-Hong Pan, Jia-Ling Yang

**Affiliations:** ^1^Department of Life Science, Institute of Biotechnology, National Tsing Hua University, Hsinchu 30013, Taiwan; ^2^Department of Pharmacodynamics and Toxicology, Botanical Drug Technology Division, Biomedical Technology and Device Research Laboratories, Industrial Technology Research Institute, Hsinchu 30011, Taiwan; ^3^Department of Chemistry, Manufacturing and Controls Technology, Botanical Drug Technology Division, Biomedical Technology and Device Research Laboratories, Industrial Technology Research Institute, Hsinchu 30011, Taiwan

## Abstract

*Alpinia oxyphylla* is a traditional Chinese medicine widely used for treating diarrhea, ulceration, and enuresis. Moreover,* A. oxyphylla* is effective for cognitive function improvement and nerve regeneration. Multiple sclerosis (MS) is a chronic neuronal inflammatory autoimmune disease that commonly affects young adults in high-latitude regions. The aim of this study was to evaluate the beneficial effects of* A. oxyphylla* in an experimental autoimmune encephalomyelitis (EAE) mouse model, which is an extensively used model for human MS. The ethanolic extract of* A. oxyphylla* fruit (AO-1) was orally administered to EAE mice. Our results showed AO-1 significantly reduced EAE symptoms. Histopathological analysis showed AO-1 reduced demyelination, inflammation, gliosis, and axonal swelling in the spinal cord. Furthermore, immunohistochemistry and quantitative polymerase chain reaction (qPCR) studies revealed that the infiltration of CD4^+^, CD8^+^ T cells, and CD11b^+^ monocytes into the spinal cord decreased in the AO-1-treated group. Mechanistically, the Th1 transcription factor T-bet, Th17 transcription factor retinoic acid receptor–related orphan receptor *γ* (ROR*γ*t), and inflammatory cytokines interferon (IFN)-*γ* and interleukin (IL)-17 were reduced in the spinal cords of mice treated with AO-1. The expression levels of T-bet and ROR*γ*t were also lowered in the spleens of those mice. Further* in vitro *study showed AO-1 inhibited production of IFN-*γ*, IL-2, and tumor necrosis factor-*α* from MOG_35-55_-peptide-stimulated splenocytes. One component isolated from AO-1, yakuchinone A, inhibited IL-17 production* in vitro* and reduced EAE symptoms in the mice. Collectively, our results indicate that AO-1 ameliorated the severity of EAE in mice and may involve the regulation of Th1/Th17 response.* A. oxyphylla* warrants further investigation, particularly regarding its clinical benefits for MS.

## 1. Introduction

Multiple sclerosis (MS) is a chronic autoimmune disease characterized by central nervous system (CNS) inflammation and demyelination, which can lead to incomplete nerve signal transmission. It is the most common demyelination disease in highly developed countries [1]. Approximately 2.3 million MS patients exist globally, and the prevalence of MS is 50–300 per 100,000 people [2]. MS can occur at any age, and most patients are diagnosed between the age of 20 and 40 years. It is a major cause of severe disability in young adults [2, 3]. The most common manifestations of MS include fatigue, pain, sensory loss, motor impairment, bladder control problems, cognitive impairment, and visual symptoms [4, 5]. In MS patients with relatively severe and complicated symptoms, MS substantially affects personal quality of life, social relationships, and productivity. MS is a costly neuroinflammatory disorder. In the United States, the cost of direct or indirect health care for an MS patient ranges from $8,528 to $52,244 annually [6]. However, no cure for MS exists currently.

The etiology of MS remains elusive. Environmental factors such as latitude, smoking, Epstein–Barr virus infection, genetic susceptibility, and immune regulation all play roles [7]. Regarding immunity, both adaptive and innate immune systems contribute to the pathogenesis of MS. Autoreactive lymphocytes raised from peripheral lymph nodes along with activated antigen-presenting cells invade the CNS and drive the initial inflammatory response. Although the pathogenesis of MS may be mediated by various immune components such as autoantibodies, the complement system, and innate immune cells, T cells are believed to have crucial roles in both initiation and chronic states [5]. The genetic factor of HLA-DR2 for the susceptibility of MS strongly implicates the involvement of CD4^+^ T cells in the pathogenesis of MS [8]. Numerous studies have supported that two subsets of CD4^+^ T cells, namely, Th1 and Th17 cells, play pivotal roles in the pathogenesis of MS. Th1 cells are characterized by the expression of the master transcription factor T-bet and production of the inflammatory cytokine interferon (IFN)-*γ*. Myelin-reactive Th1 cells are more frequently detected in the peripheral blood of MS patients than in that of healthy controls [9]. Studies conducted on the most widely used experimental autoimmune encephalomyelitis (EAE) model for MS have shown that myelin-reactive Th1 cells and cytokine IFN-*γ* are crucial in the initiation of EAE and that Th1 cells can induce EAE through adoptive transfer to naïve recipients [10–12]. Th17 cells constitute another subset of T cells that produce the inflammatory cytokines interleukin (IL)-17A, IL-22, and tumor necrosis factor (TNF)-*α* and express the transcription factor retinoic acid receptor–related orphan receptor *γ* (ROR*γ*) [13]. In the relapse phase of MS, the population of Th17 cells in cerebrospinal fluid and peripheral blood increases with high proliferation capacity [14]. In addition, mice with defective Th17 cells were reported to be resistant to EAE [15]. Altogether, the findings of these studies strongly suggest that Th1 and Th17 cells contribute to EAE and MS pathogenesis.

*Alpinia oxyphylla* Miq. belongs to the Zingiberaceae family and is widely cultivated and distributed in South China, with Hainan and Guangdong being the two main producing regions. The dry fruit of* A. oxyphylla *has commonly been used as food or medicine for centuries. It has been used as a traditional Chinese medicine for reducing saliva, alleviating stomach pain, warming the kidney, and strengthening the brain. Modern studies have shown that* A. oxyphylla* possesses a wide range of pharmacological activities, including antidiabetes, antiliver fibrosis, antidiarrheal, anticancer, and renal protection effects [16–21]. In addition, several publications have reported the neuronal protective effects of* A. oxyphylla*; for example, the total flavonoids of* A. oxyphylla *could exert antidepressant effects through a tropomyosin receptor kinase B–mediated signaling pathway [22], the herbal pair of* A. oxyphylla *and* Schisandra chinensis* could improve cognitive ability in a mouse model of Alzheimer's disease, and the n-butanol extract of* A. oxyphylla *showed protective effects on memory and learning in defect-A*β*-treated mice [23, 24]. Moreover, the water extract of* A. oxyphylla* was reported to protect neurons from ischemic damage by reducing the formation of free radicals [25]. Although these studies have demonstrated evidence of the neuroprotective effects of* A. oxyphylla*, the effects of* A. oxyphylla* in the chronic, neuronal demyelination autoimmune disease MS have never been reported.

In this study, we evaluated the beneficial effects of an ethanolic extract of* A. oxyphylla *fruit (AO-1) in a mouse EAE model. This model mimics the clinical symptoms of MS and is the most widely used animal model for human MS. Our results revealed that AO-1 could ameliorate EAE symptoms in mice, and the corresponding mechanism may involve the regulation of Th1/Th17 responses. In addition, the compound yakuchinone A isolated from AO-1 could reduce the EAE symptoms in these mice. Our findings suggest that* A. oxyphylla* has potential for further investigation on the clinical benefits of MS.

## 2. Materials and Methods

### 2.1. Chemicals and Reagents

Incomplete Freund's adjuvant was purchased from Sigma-Aldrich (St. Louis, MO, USA). Myelin oligodendrocyte glycoprotein (MOG_35-55_) peptide was purchased from Kelowna International Scientific Company (Taipei, Taiwan). Pertussis toxin was purchased from List Biological Laboratories (Campbell, CA, USA). Mycobacterium tuberculosis H37RA was purchased from Difco Laboratories (Detroit, MI, USA). Rabbit anti-mouse CD4 monoclonal, rabbit anti-mouse CD8 polyclonal, rabbit anti-mouse CD11b monoclonal, mouse anti-mouse T-bet monoclonal antibodies, and goat anti-rabbit IgG H&L (horseradish peroxidase, HRP) were purchased from Abcam (Cambridge, UK). A rat anti-mouse ROR*γ*t monoclonal antibody was purchased from Affymetrix (Santa Clara, CA, USA). A mouse anti-mouse *α*-Tubulin monoclonal antibody, goat anti-rat IgG H&L (HRP), and horse anti-mouse IgG H&L (HRP) were purchased from Cell Signaling Technology (Saint Louis, MO, USA). The RNeasy Kit and reverse transcription kit were purchased from Qiagen (Hilden, Germany). The primers used for quantitative polymerase chain reaction (qPCR) were synthesized by Mission Biotech (Taipei, Taiwan). iQ™ SYBR® Green was purchased from Bio-Rad (Hercules, CA, USA). Thiazolyl blue tetrazolium bromide (MTT), Phorbol 12-myristate 13-acetate (PMA), Ionomycin, and Cremophor® EL were purchased from Sigma-Aldrich (St. Louis, MO, USA). IL-2, IFN-*γ*, and TNF-*α* ELISA kits were purchased from R&D Systems (Minneapolis, MN, USA). Ethanol (95%) was purchased from Tobacco & Liquor Corporation (Taipei, Taiwan).

### 2.2. Preparation of AO-1

The fruits of* A. oxyphylla *were purchased from Jin Jih Bao An Trading Co. Ltd. (Taiwan), which imported them from Baisha County of Hainan Island, China. Each fruit of* A. oxyphylla *was DNA authenticated by internal transcribed spacer (ITS) sequences. The powdered fruit of* A. oxyphylla *was mixed with 95% ethanol at a ratio of 1:10 (w/v), after which extraction was conducted in an orbital shaking incubator (120 rpm) at 25°C for 7 days. After extraction, the mixture was vacuum filtered and the solid residue was discarded. The ethanol filtrate was then evaporated under reduced pressure until it achieved dryness; the product was lyophilized to obtain AO-1. The estimated yield was 139 g of AO-1 from 2.5 kg of starting materials.

### 2.3. Constituent Analysis of AO-1

Ultraperformance liquid chromatography (UPLC) was performed on a Dionex Ultimate 3000 HPLC system equipped with a binary solvent delivery system, an autosampler, and a photodiode array detection (PDA) system. The UPLC process was performed using an ACQUITY BEH C18 (1.7 *μ*m, 2.1 × 150 mm) column. The mobile phase consisted of solvent A (HCOOH: H_2_O = 0.1:100) and solvent B (HCOOH: CH_3_CN = 0.1:100). The UPLC elution condition was optimized as follows: isocratic 85% A (0–1 min), linear gradient from 85% to 50% A (1–3 min), isocratic 50% A (3–8 min), linear gradient from 50% to 5% A (8–12 min), isocratic 5% for 3 min, and back to 85% A for 3 min. The flow rate was 0.4 mL/min, column temperature was 45°C, and injection volume was 1 *μ*L. Mass spectrometry was performed on a Bruker Maxis Impact quadrupole time-of-flight (TOF) system equipped with an electrospray ionization (ESI) source operating in positive mode. The nebulization gas flow rate was set to 600 L/h at a temperature of 300°C, and the cone gas flow rate was set to 50 L/h; the source temperature was set to 100°C. The capillary voltage and cone voltage were set to 2700 and 35 V, respectively. The TOF acquisition rate was set to 0.2 s, with a 0.01 s interscan delay.

### 2.4. Animals

Female C57BL/6 mice (8 weeks of age) were purchased from the National Laboratory Animal Center (NLAC, Taiwan). The mice were housed with free access to water and food under a constant environment that was maintained at a temperature of 23 ± 2°C, relative humidity of 40%–70%, and 12:12-h light–dark cycle at the Animal Research Facility of the Industrial Technology Research Institute (ITRI, Taiwan). To ensure the health of the animals, observation and recording were performed daily by veterinarians and investigators of ITRI during the quarantine and experimental period. The experimental protocol for inducing EAE in mice was reviewed and approved by the Institutional Animal Care and Use Committee (IACUC) of ITRI.

### 2.5. EAE in Mice

Female C57BL/6 mice at 10–12 weeks of age were immunized with subcutaneous injection of 200-*μ*g MOG_35-55_ peptide and 400-*μ*g mycobacterium tuberculosis H37Ra in a 200-*μ*L emulsion. The mice received intraperitoneal injection of 500-ng pertussis toxin on Day 0 and Day 2. On Day 7 after immunization or the onset of EAE symptoms, the mice were divided into groups and treated for 14 days. The prepared AO-1 was dissolved in a solution composed of 10% DMSO, 10% Cremophor® EL, and 80% saline. Yakuchinone A was dissolved in a solution composed of 5% DMSO, 30% Cremophor® EL, and 65% saline. For the treatment of EAE, mice were orally or intraperitoneally dosed with AO-1 (300 and 1000 mg/kg) or yakuchinone A (50 mg/kg) daily. The severity and symptoms of EAE were evaluated after the mice had been grouped as follows: score 0, no sign; score 0.5, no tail movement and only transient tail lifting; score 1, tail weakness; score 2, tail paralysis and mild hind limb weakness; score 3, moderate to severe hind limb paresis or mild forelimb weakness (or both); score 4, complete hind limb paralysis or moderate to severe forelimb weakness (or both); score 5, quadriplegia with uroclepsia or moribund state; and score 6, death.

### 2.6. Histological Analysis

The mice were euthanized on Day 21 after the immunization of MOG_35-55_ peptide. Spinal cords were removed and fixed in 10% phosphate-buffered formaldehyde. Paraffin-embedded 3-*μ*m-thick spinal cord cross sections were stained with hematoxylin and eosin (H&E) to confirm tissue lesion. The other slides were stained with Luxol fast blue for examination of demyelination. Lesions were scored based on demyelination, inflammation, axonal swelling, and gliosis according to the following standard. Demyelination is a characteristic of MS and was graded from 0 to 5 as follows: 0 = no demyelination; 1 = few, scattered demyelination; 2 = small groups of demyelination; 3 = large groups of demyelination; 4 = massive demyelination over one-half of the white matter; and 5 = widespread demyelination. Inflammation is the main trigger of the events leading to CNS tissue damage and was graded from 0 to 4 as follows: 0 = no inflammatory cells; 1 = few, scattered inflammatory cells; 2 = inflammatory cells infiltrate into perivascular cuffs; 3 = perivascular cuffing with extension into parenchyma; and 4 = extensive perivascular cuffing with increasing subarachnoid and parenchymal inflammation. Axonal swelling was graded from 0 to 3 as follows: 0 = no axonal swelling; 1 = few, scattered swollen axons; 2 = small groups of swollen axons; and 3 = large groups of swollen axons. Gliosis is an astrocytic reaction to CNS damage, and it was graded from 0 to 5 as follows: 0 = no gliosis; 1 = minimal gliosis (1%–10%); 2 = slight gliosis (11%–25%); 3 = moderate gliosis (26%–50%); 4 = moderately severe gliosis (51%–75%); and 5 = severe or high gliosis (76%–100%). The severity grade was determined and reviewed by two veterinary pathologists of the National Laboratory Animal Center, Taiwan. For immunocytochemistry, the paraffin-embedded sections were deparaffinized, gradually dehydrated, and blocked for endogenous peroxidase activity with 0.5% H_2_O_2_. The sections were incubated with primary antibodies (anti-mouse CD4 antibody, at 1/1000 dilution; anti-mouse CD8 antibody, at 1/200 dilution; and anti-mouse CD11b antibody, at 1/4000 dilution), followed by HRP goat anti-rabbit secondary antibody (at 1/500 dilution). Immunopositive infiltrating cells were determined using an Olympus microscope (Olympus DT70-BX51).

### 2.7. Splenocyte Culture

For an* in vitro* assay, splenocytes were isolated from MOG_35-55_-immunized mice on Day 21. Cells were seeded into a 96-well plate at a concentration of 3 × 10^6^ cells/mL and cultured in 10% FBS RPMI1640 medium with 20 *μ*g/mL MOG_35-55_ peptide. AO-1 was added and maintained at 37°C with 5% CO_2_ for 48 h. Cell viability was determined through the MTT method. IFN-*γ*, IL-2, and TNF-*α* in supernatants were assayed using ELISA kits according the manufacturer's recommendations.

### 2.8. Western Blot Analysis

Dissected mouse spleen tissues were cut into smaller pieces and transferred to a homogenizer with lysis buffer containing protease inhibitor. The total proteins from the tissue extracts were measured using a bicinchoninic acid (BCA) protein assay kit. Equal amounts of protein were subjected to NuPAGE 4%–12% Bis-Tris Gel and were transferred to a 0.45-*μ*m nitrocellulose membrane through electroblotting. The membranes were blocked for 1 h in TBST (50 mM Tris-HCl, 150 mM NaCl, pH 7.6 with 0.1% Tween-20) containing 5% skim milk and were then immersed in primary antibodies overnight at 4°C. After washing with TBST, the membranes were incubated with HRP-conjugated anti-rabbit or anti-mouse IgG for 2 h at room temperature. The membranes were washed with TBST again, and the signal intensities of target proteins were detected by a chemiluminescent HRP substrate. The exposures were imaged by a BioSpectrum®600 imaging system (UVP, USA).

### 2.9. qPCR

Total RNAs were isolated from the frozen brains and spinal cords of the mice by using the Qiagen RNeasy Mini Kit. cDNA was synthesized with 2000 ng of extracted RNA using a Quanti®Tect reverse transcription kit, according to the manufacturer's instructions. qPCR was performed using SYBR Green Supermix. The primer sequences are listed as follows: IL-17 (forward, 5′GCTCCAGAAGGCCCTCAGACT3′; reverse, 5′ CCAGCTTTCCCTCCGCATTGA3′), IFN-*γ* (forward, 5′TGAACGCTACACACTG-CATCTTGG3′; reverse, 5′CGACTCCTTTTCCGCTTCCTGAG3′), CD4 (forward, 5′TGTGCCGAGCCATCTCTCTTAGG3′; reverse, 5′GCACTGAGAGTGTCATG-CCGAAC3′), CD8 (forward, 5′ATGCAGCCATGGCTCTGGC3′; reverse, 5′GCATG-TCAGGCCCTTCTGGGT3′), CD11b (forward, 5′GGGCACGGTGGCAGGTGAA3′; reverse, 5′GCTGGCTGTGGGAGGCACTG3′), vascular adhesion molecule-1 (VCAM-1, forward, 5′CAAGGGTGACCAGCTCATGA3′; reverse, 5′TGTGCAGCCACCTGAGATCC-3′), *α*4 integrin (forward, 5′AAGGAAGCCAGCGTTCATATT3′; reverse, 5′TCA-TCATTGCTTTTGCTGTTG3′), T-bet (forward, 5′GATCGTCCTGCAGTCTCTC-C3′; reverse, 5′AACTGTGTTCCCGAGGTGTC3′), ROR*γ*t (forward, 5′CATCTCT-GCAAGACTCATCG3′; reverse, 5′CAGGGGATTCAACATCAGTG3′), and *β*-actin (forward, 5′TGGAATCCTGTGGCATCCATGAAAC3′; reverse, 5′TAAAACGCAGCTCAGTAACAGTCCG3′). The PCR processes were performed under the following conditions: 95°C for 3 min, followed by 40 cycles of denaturation at 95°C for 10 s, annealing at 52°C for 10 s, and extension at 72°C for 30 s (CFX Real-Time PCR Detection Systems, California, USA). The relative quantification was analyzed using the 2-ΔΔCt method.

### 2.10. Anti-IL-17 Production from EL4 Cells

EL4—a mouse lymphoma cell line—cells were procured from the American Tissue Culture Collection (ATCC; Manassas, VA, USA) and cultured in RPMI1640 supplemented with 10% (v/v) fetal bovine serum, 1.5 g/L sodium bicarbonate, 1 mM sodium pyruvate, 0.12 mM NEAA, and 1 mM 2-mercaptoethanol at 37°C under 5% CO_2_. The EL4 cells were seeded in a 96-well plate at a density of 1 × 10^5^ cells/well and treated with PMA (10 ng/mL) and ionomycin (5 ng/mL) in the presence or absence of different concentrations of yakuchinone A for 16 h. Cell viability was determined using the MTT assay, and IL-17 was detected through ELISA.

### 2.11. Statistical Analysis

Data are presented as mean ± SEM (standard error of the mean). Differences between groups were analyzed using Student's two-tailed t-test. Statistical significance was defined as *P* < 0.05.

## 3. Results

### 3.1. AO-1 Ameliorates EAE Severity in Mice

**Figure 1:**
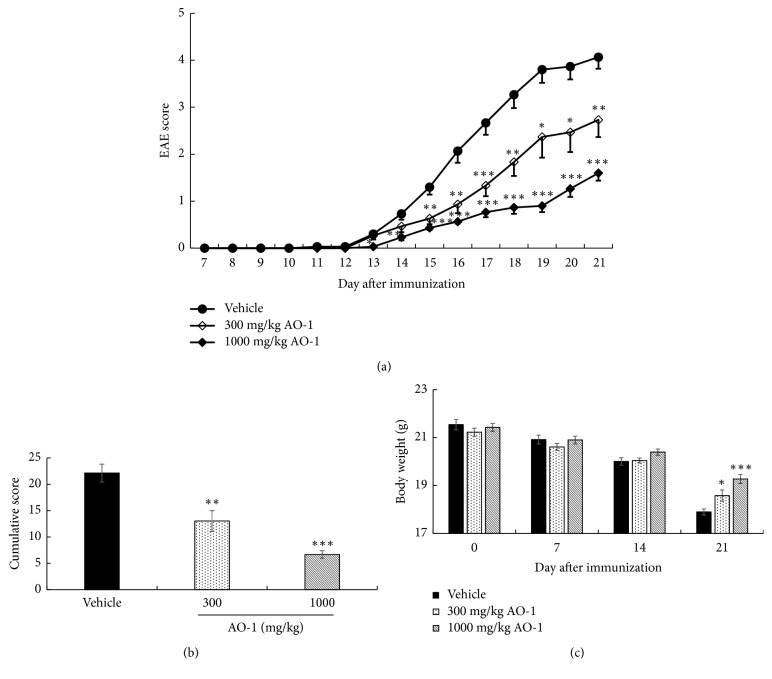
Effects of AO-1 in EAE mice. (a) EAE score, (b) cumulative score, (c) body weight. EAE was induced in C57BL/6 mice by MOG_35-55_ peptide and pertussis toxin. Mice were untreated (vehicle) or treated with 300 or 1000 mg/kg AO-1 daily on Day 7 after immunization for 14 days. EAE score and body weight were recorded daily and weekly. Data are expressed as mean ± SEM (n = 10). *∗ P *< 0.05, *∗∗ P *< 0.01, and *∗∗∗ P *< 0.001 compared with the vehicle group.

To evaluate the biological effects of AO-1, mice were orally treated with 300 mg/kg or 1000 mg/kg AO-1 on Day 7 after MOG_35-55_ peptide immunization. The daily EAE score, cumulative EAE score, and body weight were recorded. The signs of EAE progressively increased after Day 12 in the vehicle group, and the mean EAE scores were over 2 and 3 on Day 16 and Day 18, respectively. The mean EAE score was 4.07 ± 0.25 on Day 21 prior to termination of the study. In mice treated with 300 mg/kg AO-1, the signs of EAE exhibited delayed onset relative to the vehicle group. The mean EAE score was 2.73 ± 0.37 at the end of the study. In the group of mice treated with 1000 mg/kg AO-1, the EAE progress was obviously decreased and the EAE score, symptoms, and neurological disorders were significantly attenuated. The final EAE score decreased to 1.60 ± 0.16 (Figure 1(a)). In addition, the entire EAE burden in each group throughout the experiment was expressed as a cumulative score, which was the sum of the daily score of each mouse. Our results showed that the cumulative scores decreased significantly in a dose-dependent manner in mice fed with AO-1 compared with the vehicle group (Figure 1(b)). In general, after EAE induction, the body weight gradually decreases and EAE symptoms appear. As illustrated in Figure 1(c), the data indicated that AO-1 treatment prevented body weight loss significantly on Day 21 relative to untreated vehicle group. In particular, no toxicity signs were evident in the AO-1-treated groups throughout the experiment.

### 3.2. AO-1 Reduces Demyelination and Inflammation in EAE Mice

**Figure 2:**
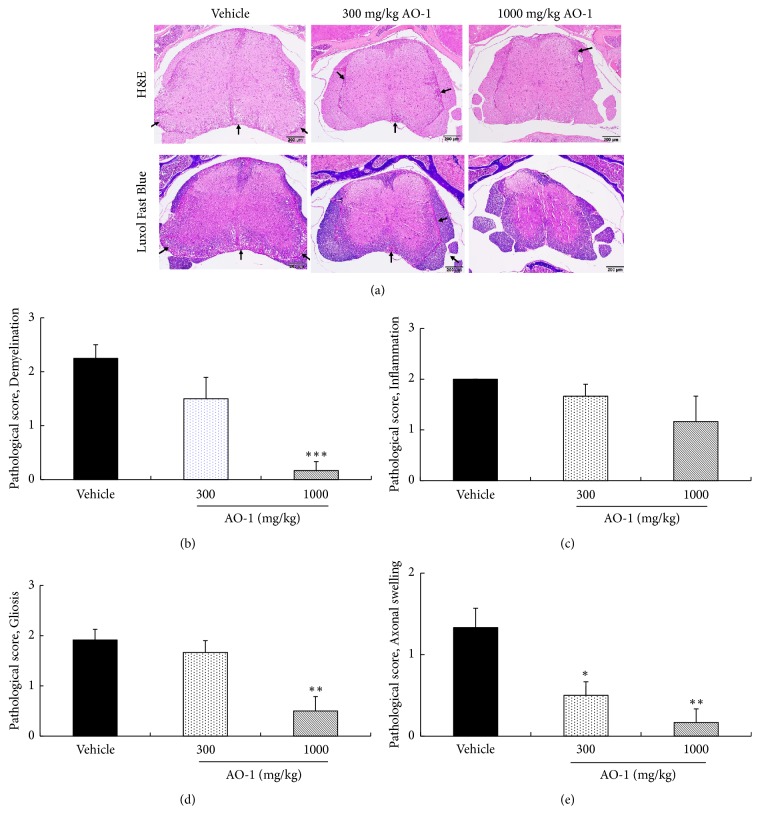
AO-1 ameliorates histopathology of EAE mice. (a) H&E and Luxol fast blue staining. (b) Pathological score of demyelination, (c) pathological score of inflammation, (d) pathological score of gliosis, and (e) pathological score of axonal swelling. On Day 21 after immunization, spinal cords of EAE mice were harvested and then subjected to H&E and Luxol fast blue staining. Demyelination, inflammation, gliosis, and axonal swelling were scored semiquantitatively as described in the Materials and Methods section. Arrow indicates demyelination areas accompanied by inflammation. Data are expressed as mean ± SEM (n = 4). *∗ P *< 0.05, *∗∗ P *< 0.01, and *∗∗∗ P *< 0.001 compared with the vehicle group.

To further confirm the beneficial effects of AO-1, the spinal cords of untreated and AO-1-treated EAE mice were harvested. We applied H&E staining to assess lesion severity, and we applied Luxol fast blue staining to evaluate demyelination. Histologically, the mice in the vehicle group developed moderate to severe EAE in different segments of the spinal cord, characterized by multifocal areas of myelin loss within the white matter of the spinal cord along with variable degrees of nonsuppurative inflammation, gliosis, and axonal injury (Figure 2(a)). After AO-1 administration, the lesion severity, distribution, and incidence of EAE in the spinal cord were ameliorated. The histological features of demyelination, inflammation, gliosis, and axonal swelling were reduced in the group of mice treated with 1000 mg/kg AO-1 (Figures 2(b)–2(e)). Collectively, these results support the ameliorative effects of AO-1 on the paralysis symptoms of EAE mice.

### 3.3. AO-1 Reduces the Infiltration of Immune Cells into the Spinal Cord and Brain

**Figure 3:**
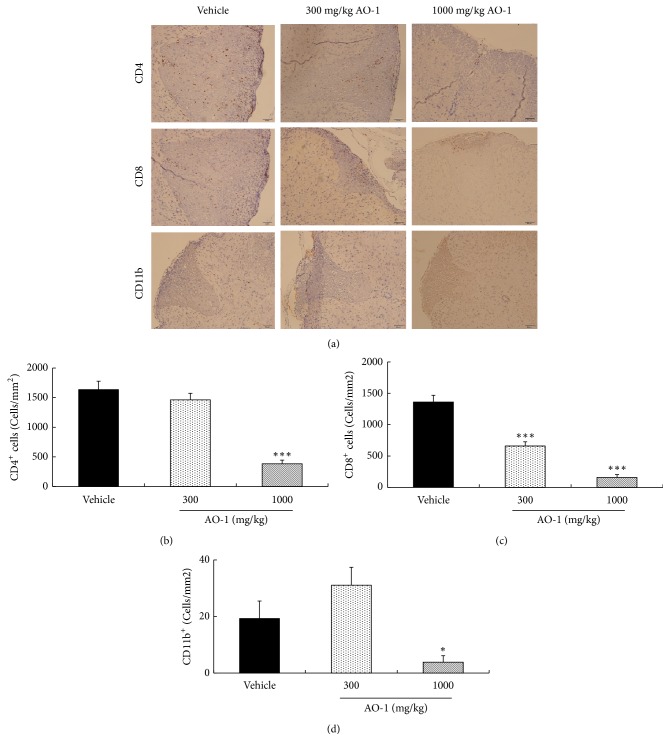
Effects of AO-1 on the infiltration of CD4^+^, CD8^+^ T cells, and CD11b^+^ monocytes in the spinal cord of EAE mice. (a) IHC analysis of untreated vehicle and AO-1-treated groups. (b)–(d) Quantitation of CD4^+^, CD8^+^ T cells, and CD11b^+^ monocytes. On Day 21 after MOG_35–55_ immunization, the infiltrated T cells and monocytes in the spinal cord of EAE mice were measured through IHC analysis using specific antibodies. The numbers of CD4^+^, CD8^+^ T cells, and CD11b^+^ monocytes were quantified. Data are presented as mean ± SEM (n = 4). *∗ P* < 0.05 and *∗∗∗ P *< 0.001 compared with vehicle group. Scale bars, 50 *μ*m.

**Figure 4:**
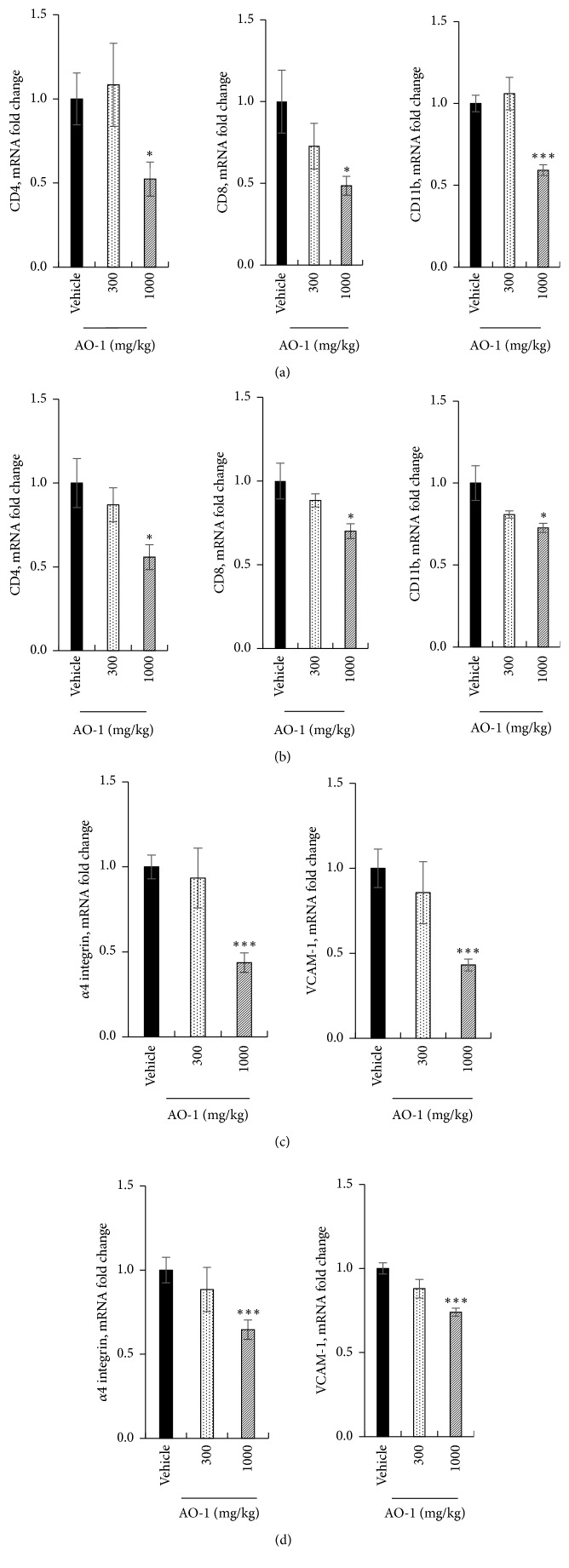
Effects of AO-1 on mRNA expression in EAE mice. (a), (c) Spinal cord. (b), (d) Cerebral cortex. On Day 21 after MOG_35–55_ immunization, the spinal cords and cerebral cortices of untreated vehicle and AO-1-treated mice were harvested and the mRNAs expression levels were analyzed through qPCR. Date are expressed as mean ± SEM (n = 4). *∗ P *< 0.05 and *∗∗∗ P *< 0.001 compared with vehicle group.

Next, we evaluated the effects of AO-1 on immune cell infiltration into the CNS in EAE mice. The spinal cord sections were assessed through immunohistochemistry (IHC) analysis. As illustrated in Figure 3, the results showed that in the mice treated with 1000 mg/kg AO-1, the infiltrated CD4^+^ T cells, CD8^+^ T cells, and CD11b^+^ monocytes were significantly reduced relative to the vehicle group (Figures 3(a)–3(d)). Furthermore, we analyzed the mRNA expression of surface markers by performing qPCR. Our results revealed that CD4, CD8, and CD11b were noticeably inhibited in the spinal cord and cerebral cortex in the group treated with 1000 mg/kg AO-1 (Figures 4(a) and 4(b)). During the initiation and chronic phases of MS, the critical step is the infiltration of leukocytes into the CNS. The binding of *α*4*β*1 integrin to its ligand VCAM-1 is the key factor for the adhesion and infiltration of antigen-specific lymphocytes into the CNS. We next examined the effects of AO-1 on the expression of the adhesion molecules *α*4 integrin and VCAM-1 in the spinal cord and cerebral cortex of EAE mice. Our data showed that the mRNA expression levels of both adhesion molecules were reduced significantly in the spinal cord and cerebral cortex of the mice treated with 1000 mg/kg AO-1 (Figures 4(c) and 4(d)). These results imply that AO-1 significantly reduced the infiltration of leukocytes into the brain and spinal cord.

### 3.4. AO-1 Alleviates Th1/Th17 Response

**Figure 5:**
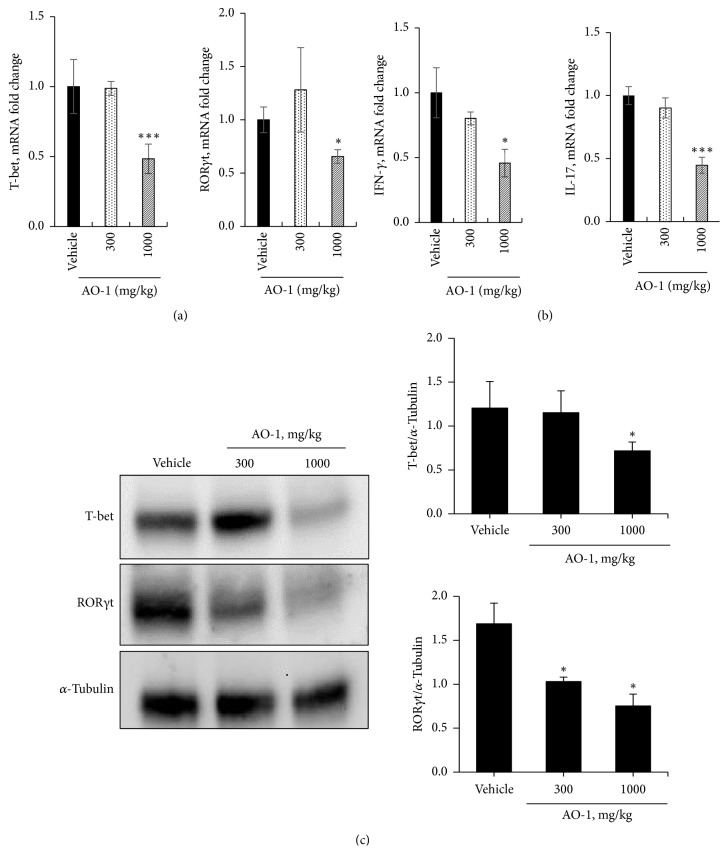
Effects of AO-1 on Th1 and Th17 cell regulation. (a) mRNAs expression of T-bet and ROR*γ*t in the spinal cord, (b) mRNAs expression of IFN-*γ* and IL-17 in the spinal cord, and (c) protein expression levels of T-bet and ROR*γ*t in the spleen. On Day 21 after MOG35–55 immunization, the spinal cords and spleens of untreated vehicle and AO-1-treated mice were harvested. The mRNA and protein expression levels were analyzed through qPCR and Western blot analysis. Data are expressed as mean ± SEM (n = 4). *∗ P *< 0.05 and *∗∗∗ P* < 0.001 compared with vehicle group.

**Figure 6:**
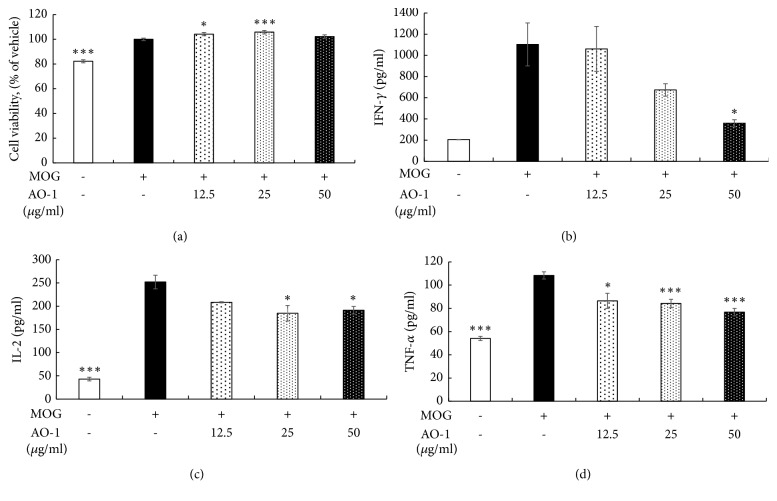
Effects of AO-1 on inflammatory cytokine production in MOG_35-55_-peptide-stimulated splenocytes. (a) Cell viability. (b) IFN-*γ*, (c) IL-2, and (d) TNF-*α* expression. C57BL/6 mice were immunized with MOG_35-55_ peptide and splenocytes were isolated after 21 days. Splenocytes were treated with MOG_35-55_ peptide and AO-1 for 48 h. Cell viability was assayed using the MTT method, and cytokine production was determined through ELISA. Date are expressed as mean ± SEM. *∗P *< 0.05 and *∗∗∗P* < 0.001 compared with vehicle group.

We further explored the possible mechanism involved in the biological effects of AO-1. The expression levels of the Th1-specific transcription factor T-bet and Th17 master transcription factor ROR*γ*t in the spinal cord were analyzed using qPCR. As illustrated in Figure 5, the mRNA levels of T-bet and ROR*γ*t decreased in the group treated with 1000 mg/kg AO-1. In addition, the mRNA levels of the inflammatory cytokines IFN-*γ* and IL-17 decreased in the group of mice treated with 1000 mg/kg AO-1(Figures 5(a) and 5(b)). The protein expression levels of T-bet and ROR*γ*t in the spleen also decreased in the group of mice treated with 1000 mg/kg AO-1 (Figure 5(c)). To further evaluate the effects of AO-1 on T-cell activation, we conducted an* in vitro* culture of splenocytes. Splenocytes were isolated from EAE mice and then treated with MOG_35–55_ peptide with or without AO-1 for 48 h. Cell viability was determined using the MTT method, and the supernatant was harvested for cytokine analysis through ELISA. Our results demonstrated that under the noncytotoxic conditions, AO-1 significantly reduced the expression levels of the inflammatory cytokines IFN-*γ*, IL-2, and TNF-*α* (Figure 6). Collectively, these results indicate that AO-1 ameliorated EAE in mice, and this may have occurred through the regulation of Th1/Th17 response.

### 3.5. Phytochemical Constituents of AO-1

**Figure 7:**
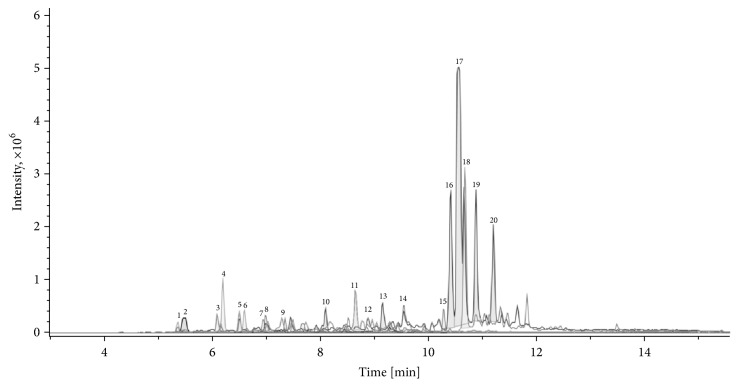
Chromatographic fingerprint of AO-1, performed by UPLC-ESIMS. Twenty peaks with high intensity were sorted and further analyzed.

We analyzed the constituents of AO-1 by using UPLC-ESIMS. The fingerprint of AO-1 exhibited 20 major peaks with high intensity (Figure 7). These peaks were further sorted and analyzed according to the molecular weight data from the Reaxys database and from the literature [26]. The results indicated that AO-1 contained sesquiterpenes (such as oxyphyllenodiol A, oxyphyllol D, and oxyphyllone D) and diarylheptanoids (such as yakuchinone A), as presented in Table 1.

### 3.6. Identification of One Active Component of AO-1

**Figure 8:**
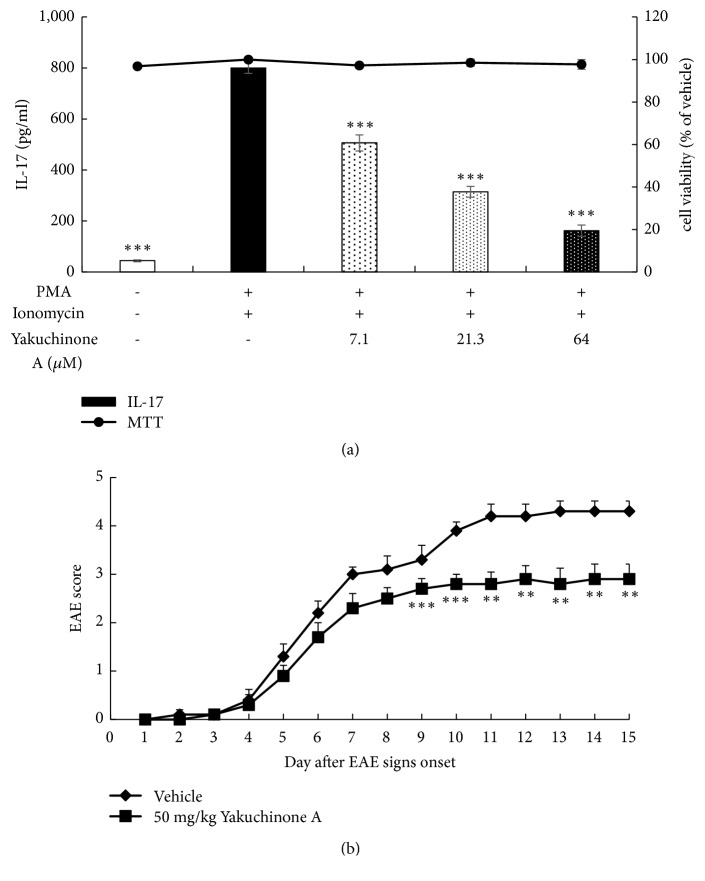
Anti-IL-17 production activity and EAE ameliorative effects of yakuchinone A. (a) Anti-IL-17 production activity. PMA and ionomycin-activated EL4 cells were treated with yakuchinone A overnight. Cell viability was assayed using the MTT method and IL-17 concentration was determined through ELISA. (b) EAE score. EAE was induced in C57BL/6 mice by MOG_35-55_ peptide and pertussis toxin. Mice were untreated (vehicle) or treated with 50 mg/kg yakuchinone A intraperitoneally. EAE scores were evaluated daily. Data are expressed as mean ± SEM (n =10). *∗∗ P* < 0.01 and *∗∗∗ P* < 0.001 compared with vehicle group.

To reveal the possible active constituents of AO-1, we used* in vitro* bioassay-guided fractionation and purified one diarylheptanoid, yakuchinone A. The anti-IL-17 production activity of yakuchinone A was evaluated in mouse lymphoma EL4 cells. In the concentration without cytotoxicity, yakuchinone A reduced IL-17 production in a dose-dependent manner. The IC_50_ value was 11.5 *μ*M (Figure 8(a)). Additionally, we tested the effects of yakuchinone A in EAE mice. Mice were treated with 50 mg/kg yakuchinone A intraperitoneally daily on the day that signs of ataxia appeared. Our results revealed that yakuchinone A showed a significant EAE score reduction from Day 9 to Day 15 after EAE onset. This result suggests that yakuchinone A was one active component of AO-1 (Figure 8(b)).

## 4. Discussion

MS is an inflammatory demyelination disease. Most patients usually experience relapse and remission episodes, gradually progress to a severe condition, and eventually develop a long-term disability. No cure for MS exists currently. Current treatments focus on relieving symptom or reducing inflammation caused by demyelination. The safety of long-term use of clinical drugs for MS is still a major concern. New medication with high efficacy and safety is still an urgent and unmet medical need.

Animal models are critical tools to unravel the pathogenesis of human diseases and translate the preclinical results into human clinical usage. Although no animal models can recapitulate all characteristics of human diseases, EAE is the most widely accepted model for human MS. Previous publications have shown that most elements of MS pathogenesis have conformed to EAE models. The US Food and Drug Administration approvals of therapeutic drugs such as fingolimod, dimethyl fumarate, and laquinimod all have been validated by EAE studies [27, 28]. EAE was first developed in monkeys in 1933 [29] and can be induced in various species currently. Due to the ease of access and operation, mouse models constitute the most common models for EAE [28]. In this study, we induced EAE in C57BL/6 mice; AO-1 ameliorated mouse EAE symptoms significantly in a dose-dependent manner (Figure 1). Moreover, histopathological analysis and qPCR revealed that AO-1 reduced demyelination and the infiltration of CD4^+^, CD8^+^, CD11b^+^ leukocytes into the spinal cord (Figures 2–4). These data support the ameliorative effects of AO-1 in EAE and indicate the potential of* A. oxyphylla* for MS treatment.

EAE involves predominant T-cell-mediated responses due to the initiation and progression of an immune response to myelin antigens such as myelin basic protein (MBP), proteolipid protein, and MOG [30]. Furthermore, adaptive transfer of myelin-reactive T cells to naïve recipient animals can induce EAE [31, 32]. In the context of EAE, infiltrated CD4^+^ T cells are activated by antigen-presenting cells that cause the production of inflammatory cytokines. More peripheral inflammatory cells are attracted into the CNS and further fuel the inflammation. Th1 and Th17 cells are the two main CD4^+^ T-cell subtypes implicated in EAE. When the local cytokine environment contains IFN-*γ* and IL-12, naïve CD4^+^ T cells express the transcription factor T-bet and further differentiate into Th1 cells. These cells are characterized by secretion of IFN-*γ*, IL-2, and TNF-*α*; these activate macrophages and contribute to cell-mediated immunity [33, 34]. In EAE, Th1 cells have long been assumed to play a principal role in the initiation of EAE pathogenesis [10, 11, 35]. Furthermore, mice that are deficient in T-bet were reported to be protected from EAE [36]. Recently, Th17 cells have been implicated in EAE [37]. Th17 cells are differentiated in the presence of IL-6 and TGF-*β* and express the master transcription factor ROR*γ*t. In particular, IL-23 is required for the terminal maturation of Th17 cells. This lineage CD4^+^ T cell produces unique cytokines IL-17A and IL-22, which play roles in inflammation pathogenesis and against extracellular fungi and bacterial infection [13]. Mice deficient in p19, a subunit of IL-23, were reported to be resistant to the development of EAE [38]. Additionally, adaptive transfer of IL-23-expanded Th17 cells was revealed to be highly encephalogenic in naïve recipient mice [39]. These data suggest the critical role of Th17 cells in the development of EAE. Moreover, EAE could be induced in naïve mice injected with MOG_35-55_-peptide-activated splenocytes isolated from EAE mice. The cumulative EAE scores were even higher than the scores obtained for mice subjected to active MOG_35-55_ peptide immunization [40]. Similarly, adoptive transfer of MBP-sensitized spleen cells could induce consistent EAE in rats [41]. These results imply the crucial role of splenocytes in rodent EAE models of human MS. In our study, the mRNA transcripts and protein expression of T-bet and ROR*γ*t were reduced in mice treated with 1000 mg/kg AO-1. In addition, IFN-*γ* and IL-17 were significantly reduced in RNA levels in the spinal cord (Figure 5). Furthermore, AO-1 reduced the production of the inflammatory cytokines IFN-*γ*, TNF-*α*, and IL-2 in MOG_35-55_ peptide, which stimulated splenocytes significantly (Figure 6). Collectively, our data reveal that AO-1 may regulate the Th1/Th17 response in EAE.

*A. oxyphylla* has a long clinical history in China and is widely cultured in southern provinces of China such as Hainan, Yunnan, and Guangxi. The traditional medical functions of* A. oxyphylla*, which include tonifying the kidney, reducing urine, reinforcing the spleen, reducing pain, dispelling cold, and strengthening the brain, are recorded in many Chinese folk pharmacopeias. Among these traditional medical applications, we were interested in the application of* A. oxyphylla* in neuron-related disorders. Extant studies have shown the neuroprotective potential of different preparations of* A. oxyphylla*; for example, studies have demonstrated the potential neuroprotective effects of n-butanol and chloroform extracts against learning and memory impairment in A*β*-induced Alzheimer's disease mice [23, 42], neuroprotective effect of an ethanolic extract against dopaminergic neurons in zebrafish, and the antioxidant activities of relevant extracts [43, 44]. Water extracts protect neurons from ischemia-induced cell death [25]. Due to these results regarding the neuroprotective biological activities of* A. oxyphylla*, we further evaluated the effects of AO-1 in EAE mice. Our data reveal that AO-1 reduced EAE symptoms significantly and ameliorated the corresponding histopathology in the spinal cord (Figures 1 and 2). These results not only echo the ancient claims of the brain-strengthening function of* A. oxyphylla* but also raise a new possibility of* A. oxyphylla* for human MS.

Numerous compounds have been isolated and identified from* A. oxyphylla* and can be categorized into diarylheptanoids, terpenes, flavones, nucleobases, and steroids [26]. Yakuchinone A and oxyphyllacinol are the major diarylheptanoid of* A. oxyphylla*, and the average content of yakuchinone A from different regions is 3.9 mg/g [45]. Previous studies have shown that yakuchinone A exhibits antioxidant and antitumor activities and attenuates COX-2, iNOS, and TNF*α* production in TPA-stimulated mouse skin [46, 47]. To explore the possible active constituents of AO-1, we used a bioassay-guided fractionation method and further purified yakuchinone A from* A. oxyphylla*. Yakuchinone A inhibited IL-17 production and decreased the progression of EAE in the late phase (Figure 8). This finding suggests that yakuchinone A is one of the active ingredients of AO-1 and that the beneficial effects of AO-1 in EAE may arise from a series of compounds. In addition to diarylheptanoids, AO-1 contained sesquiterpenoids (Table 1). According to publications, sesquiterpenoids are the major components of volatile oil from the fruits of* A. oxyphylla*; additionally, nootkatone exists abundantly in the oil, and it can inhibit NO products and anti-*β*-hexosaminidase activity. Furthermore, sesquiterpenoids such as oxyphyllol A and isocyperol reduce inflammation by reducing NO production [48]. Tectochrysin, chrysin, kaempferol, rhamnocitrin, and pinocembrin are flavones isolated from* A. oxyphylla.* These compounds offer versatile biological activities such as antioxidant and anti-inflammation activities [49]. Based on these publications, compounds with anti-inflammation and antioxidant activities may have the potential to halt the progression of EAE. Additional studies are warranted to reveal the chemical constituents that contribute to the biological activity of AO-1.

## 5. Conclusions

In summary, our study showed that AO-1 alleviated EAE in mice and that one of the biological functions of AO-1 may involve the regulation of Th1 and Th17 cells. Yakuchinone A was one component isolated from AO-1 that reduced IL-17 production* in vitro* and ameliorated EAE in mice. Based on our results and the experiences of Chinese folk medicine,* A. oxyphylla* warrants further investigation, particularly regarding its clinical benefits for neuronal diseases, including human MS.

## Figures and Tables

**Table 1:** Possible constituents of AO-1 deduced from references by molecular formula.

[M+H]^+^	Molecular formula	Peak number, RT(min)	Possible constituents
239.1653	C_14_H_22_O_3_	1 (5.4)	oxyphyllendiol B, oxyphyllendiol A
253.1810	C_15_H_24_O_3_	2 (5.5)	11(S)-nootkatone-11,12-diol, 11(R)-nootkatone-11,12-diol, oxyphyllol D
219.1390	C_14_H_18_O_2_	3 (6.1)	oxyphyllone D, oxyphyllone G
195.1389	C_12_H_18_O_2_	4 (6.2), 6 (6.6)	teuhetenone A, (4S, 5E, 10R)-7-oxo-tri-nor-eudesm-5-en-4-ol
235.1702	C_15_H_22_O_2_	5 (6.5), 9 (7.3), 11(8.7),	1-hydroxycyperone, 13-hydroxy-nootkatone, oxyphyllol B, 9-hydroxy-nootkatone, 7-epiteucrenone, teucrenone, 7-epiteucrenone B
221.1541	C_14_H_20_O_2_	7 (6.9)	oxyhylladiketone, oxyphyllone C
237.1856	C_15_H_24_O_2_	8 (7.0), 12 (8.9), 14 (9.6)	11-hydroxy-1(10)-valencen-2-one, humulene 2,3:6,7-diepoxide, oxyphyllanene E, 9-hydroxyepinootkatol
223.1694	C_14_H_22_O_2_	10 (8.1), 13 (9.2)	pubescone
313.1792	C_20_H_24_O_3_	15 (10.3)	yakuchinone A
219.1743	C_15_H_22_O	16 (10.4), 17 (10.5), 19 (10.9), 20 (11.2)	(+)-nootkatone, germacrone, zerumbone, eudesma-4,11-dien-3-one, 7-epi-cyperone, *β*-cyperone, *α*-cyperone
203.1792	C_15_H_22_	18 (10.7)	nootkatene

## Data Availability

The data used to support the findings of this study are included within the article.
